# Political participation and grassroots governance: A social work perspective based on CGSS 2021 data

**DOI:** 10.1371/journal.pone.0337520

**Published:** 2025-12-05

**Authors:** Xiao Zhang, Dana Burkhanova, Yi Yang, Jingwei Tian

**Affiliations:** 1 AL-Farabi Kazakh National University, Almaty, Kazakhstan; 2 Nazarbayev University, Astana, Kazakhstan; 3 College of Philosophy and Sociology Jilin University Chang Chun, China; Federal University Otuoke, NIGERIA

## Abstract

This study, based on the 2021 China General Social Survey (CGSS), examines how different forms of political participation–spanning behavior, attitude, and affiliation –shape perceptions of grassroots governance effectiveness in safety, convenience, and social atmosphere. Framed within the conceptual lens of social work, which emphasizes community engagement and service-oriented participation, the study uses multinomial ordered logistic regression and uncovers three key insights.

First, participatory initiatives facilitated through social work–related community contexts amplify collective confidence in safety and atmosphere but paradoxically undermine convenience evaluations, revealing a procedural legitimacy paradox when community services fall short. Second, strong political attitudes cultivated through community-based outreach uniformly predicts satisfaction with safety and atmosphere, functioning as a critical accountability mechanism, especially among urban women engaged in local support programs. Third, familial political ties intersect with community networks to show generational asymmetry: paternal affiliations, often reinforced by male-led committees, predict atmosphere perceptions, while maternal ties channeled through women’s groups diminish convenience satisfaction, reflecting entrenched authoritarian familism. Control variables reveal that education predicts convenience perceptions but erodes safety and atmosphere evaluations, as more educated citizens identify gaps between policy intent and delivery; urban residency also polarizes governance expectations through differential access to community engagement platforms. Stable demographic patterns emerge, with gender and household registration maintaining consistent directional effects.

To address these dynamics, we propose differentiated participation ecosystems that leverage social work principles and community networks: (1) education–governance dialogues mediated by community practitioners; (2) voting–service audits coordinated through civic coalitions; (3) citizen panels empowering skeptical urban women; and (4) familial governance scaffolding through social work alliances, leveraging paternal heritage programs and matrifocal budget reallocation. These strategies transform social work–based engagement into a precision tool for governance optimization across urban–rural, gender, and generational divides.

## Introduction

In China’s evolving governance landscape, political participation–manifested through behavior, attitude, and affiliation – has become a critical driver of grassroots governance effectiveness, shaping how citizens perceive safety, convenience, and community atmosphere [1,2]. While existing research underscores political engagement’s role in democratic contexts, less is known about its multidimensional impacts in China, where community-based initiatives often serve as platforms resembling social work functions that mediate voting behavior, ideological attitudes, and familial political ties [3,4]. Recent policy shifts, such as the emphasis on “social governance innovation” [1] and “whole-process democracy” [2], further highlight the need to understand how participatory activities grounded in social work principles influence public perceptions at the grassroots level.

Despite growing interest, two gaps persist. First, prior research often treats political participation as a monolithic concept, overlooking how community-based engagement channels–many aligned with social work’s participatory and service-oriented ethos–shape behavioral, attitudinal, and relational dimensions of political involvement [5]. Second, few studies empirically examine how these dimensions interact with sociodemographic factors (e.g., gender, education, household registration) to shape governance outcomes in China’s varied local contexts [6,7]. Using nationally representative CGSS 2021 data, this study investigates three questions: (1) How do different forms of political participation, including community volunteering and advocacy activities inspired by social work principles, affect perceptions of safety, convenience, and community atmosphere? (2) What roles do education and urbanization, both of which shape access to participatory resources, play in these relationships? (3) How can these findings inform China’s ongoing governance modernization?

By framing social work as a conceptual lens rather than an empirical variable, this research interprets political participation as both a conduit and catalyst for community-oriented engagement. The analysis reveals how participation operates as a constellation of intersecting mechanisms–voting initiatives boost collective confidence, critical attitudes drive accountability, and familial affiliations preserve cultural continuity. These insights offer policymakers concise, evidence-based strategies anchored in community practice to optimize governance design for China’s diverse population.

The rest of this paper is organized as follows. In next Section, we review related work and hypotheses in this work. Then, the materials and methods used in this research are comprehensively reviewed in Section Materials and methods. Section Results presents detail results and analysis. In Section Discussions, we discuss potential influence and implication based this research. We conclude our study in Section Conclusions. Finally, the final section is acknowledgment for editors and reviewers.

## Related work

Political participation is a multifaceted concept without a single, universally accepted definition, encompassing a range of citizen interactions with political institutions and processes including three interrelated dimensions: behavioral acts such as voting, attitudinal orientations like beliefs about free speech, and formal affiliations with political organizations, which jointly shape citizens’ evaluations of governance quality [8–10].

Early research on perceptions of local governance effectiveness prioritized institutional performance metrics and socioeconomic determinants, such as education and corruption, over individual civic actions [11–13]. However, a growing body of work highlights that higher electoral turnout and more frequent local voting are associated with increased trust in municipal authorities and enhanced evaluations of governmental responsiveness [14,15]. Citizens who vote in local elections are more likely to report satisfaction with public service delivery and institutional performance, suggesting that participatory acts reinforce perceptions of accountability [4,16]. Furthermore, Political efficacy, an individual’s belief in their capacity to influence political outcomes, strongly predicts satisfaction with grassroots governance, as efficacious citizens hold authorities to higher standards and engage more critically with policy performance [17,18]. Moreover, attitudes favoring unrestricted public criticism of government correlate with lower tolerance for administrative shortcomings, indicating that support for free expression fosters more exacting governance evaluations [17,19,20]. Additionally, partisan affiliation further modifies these perceptions, as individuals tend to interpret policy outcomes through their ideological commitments, affecting judgments of local governance quality [21,22]. Ideological congruence between an individual’s party identity and ruling bodies leads to more favorable assessments of government effectiveness [23,24]. In the Chinese context, empirical analyses demonstrate that villagers’ willingness to engage in community elections and committee meetings correlates positively with their satisfaction with neighborhood public services, and formal party membership distinguishes citizens’ satisfaction levels with community services, reflecting identity-based biases in grassroots governance perceptions [4,13].

Therefore, political participation research spans behavioral components, attitudinal dimensions, and formal affiliations as joint determinants of governance evaluations [9,25]. International development agencies such as United Nations Development Programme (UNDP) advocate inclusive citizen engagement as a means to strengthen local accountability and service effectiveness, while the World Bank underscores digital tools’ potential to expand participatory governance and improve perceptions of administrative legitimacy [16,26,27]. Despite this progress, few studies have systematically integrated political behavior, attitudes, and affiliation within a single analytical framework to assess their impact on grassroots governance perceptions [13]. Moreover, as national conditions diverge, researchers frequently emphasize factors most relevant to their domestic environment when selecting analytical parameters. Thirdly, existing literature demonstrates limited scholarly attention to systematically investigating grassroots social governance efficacy through a comprehensive framework integrating safety, convenience, and social atmosphere as core evaluative dimensions. To address these gaps, the present study employs the 2021 Chinese General Social Survey (CGSS) to examine how political behavior, attitudes, and affiliation jointly shape citizens’ perceptions of safety, convenience, and social atmosphere under grassroots governance.

Based on aforementioned content, it leads to three hypotheses:

- Hypothesis 1: Political behavior significantly influences citizens’ perceptions of grassroots governance effectiveness.
- Hypothesis 2: Political attitude significantly influences citizens’ perceptions of grassroots governance effectiveness
- Hypothesis 3: Political affiliation significantly influences citizens’ perceptions of grassroots governance effectiveness

## Materials and methods

### Dataset

In this study, we analyze data from the 2021 Chinese General Social Survey (CGSS), China’s first nationwide, ongoing social survey project launched in 2003 by Renmin University in collaboration with research institutions across the country [28–30]. Each year, the CGSS employs a rigorous multi-stage, stratified random sampling method to interview more than 10,000 households, urban and rural alike, in all 31 provinces, autonomous regions, and municipalities. The survey’s extensive questionnaire captures information at multiple levels (societal, community, family, and individual), with dedicated modules capturing respondents’ political participation (e.g., voting behavior, attendance at public consultations, engagement in community deliberations) and their perceptions of local governance. By tracking long-term social changes and addressing key theoretical and practical questions, the CGSS promotes open data sharing, informs government policy, and supports cross-national comparisons. Leveraging this rich resource, our study investigates how distinct modes of citizens’ political participation shape their perceptions of grassroots governance effectiveness at the local level in China. In line with our predefined criteria, the 2021 CGSS dataset was rigorously screened to exclude implausible responses and observations with missing values on key variables, yielding a final analytic sample of 1790 cases.

#### Modeling.

The primary objective of this study is to investigate how citizens’ perceptions of grassroots governance effectiveness relate to our key predictors. The ordinal outcome Y (grassroots governance effectiveness in one of its three dimensions: safety, convenience or social atmosphere, each coded 1 = “strongly disagree", 2 = “disagree", 3 = “neutral", 4 = “agree", 5 = “strongly agree") and our set of predictors is modeled via a multinomial ordered logistic regression model for each dimension [31–34]. The general form of the model is expressed as follows (Formula 1):

$$Logit\left[Pr(Y_{i}\leq j)\right]=\alpha_{j}-\sum \beta_{k}x_{ki},$$ (1)

where *Y*_*i*_ is the ordinal response for respondent *i*, j∈{1,2,3,4} indexes the two cut–points (thresholds) dividing the three outcome categories, $Pr(Y_{i}\leq j)$ is the cumulative probability of being in category from 1 to *j*, $\alpha_{j}$ are the two threshold parameters (intercepts), *x*_*ki*_ are the set of explanatory variables including control and independent ones, $\beta_{k}$ are the corresponding set of coefficients. Under this specification, the same coefficient set $\beta_{k}$ is used across the thresholds, yielding a proportional-odds interpretation: a positive $\beta_{k}$ implies that higher values of *x*_*ki*_ are associated with greater odds ratio (OR) of reporting a more favorable perception of grassroots governance. Equivalently, it can be written as follows (Formula 2):

$$Log\frac{Pr(Y_{i}=j)}{Pr(Y_{i}=j+1)}=\theta_{j}+\sum \beta_{k}x_{ki},$$ (2)

where $\theta_{j}=\alpha_{j}-\alpha_{j-1}$. Thus, this model assumes proportional odds (i.e. the $\beta_{k}$ coefficients do not depend on *j*).

### Variable selection

#### Control variables.

Based on a comprehensive review of relevant literature and past research practices, and taking into account the specific context of our study, we selected three control variables [13,28,35,36]: (1) Gender, categorized as a nominal variable (1 for male, 2 for female); (2) household registration status, also treated as a nominal variable (1 for agricultural type, 2 for non-agricultural one); and (3) Education level, coded as an ordinal variable ranging from 1 to 5 (1 = no formal education, 2 = primary school, 3 = junior high school, 4 = high school, 5 = college or above).

#### Dependent variables.

Our study focuses on grassroots governance effectiveness as the dependent variable, which is conceptualized through three dimensions: safety, convenience, and social atmosphere.

To assess the safety aspect, we draw on question E36_D from the CGSS 2021 survey: “I feel that the place where I live is very safe.” Participants who selected “strongly agree" or “agree" were considered satisfied with local security conditions (coded as 5 and 4); those who chose “neither agree nor disagree" were viewed as neutral (coded as 3); and those who answered “disagree" or “strongly disagree" were interpreted as dissatisfied (coded as 2 and 1). The convenience aspect was initially designed to include three questions: whether the area is suitable for exercise (E36_A), whether fresh fruits and vegetables are easily accessible (E36_B), and whether public facilities are adequate (E36_C). However, in the final analysis, only E36_C (“whether public facilities are adequate”) was used to represent the convenience dimension. This decision was made because the responses to E36_A and E36_B were extremely unbalanced, with more than 80% of participants selecting the same option, making these items statistically uninformative. In contrast, E36_C showed a more reasonable distribution across response categories (strongly disagree: 148 (8.27%), disagree: 549 (30.67%), neutral: 159 (8.88%), agree: 677 (37.82%), strongly agree: 257 (14.36%)). Therefore, E36_C was used as the representative indicator for the convenience dimension in our analysis. For the social atmosphere, we refer to E36_E (“Neighbors care about each other") and E36_F (“Neighbors are willing to help when I need it"), which reflect the degree of mutual support and community cohesion. For both the convenience and social atmosphere questions, responses were coded using the same scheme as for safety. Together, these indicators offer a comprehensive view of how citizens perceive the effectiveness of local governance.

#### Independent variables.

Political participation refers to the ways in which citizens engage in political life to express opinions, influence decision-making, and contribute to public affairs at various levels [13,35,37–40]. It encompasses both observable behaviors and underlying attitudes that reflect individuals’ civic engagement and sense of responsibility. Political behavior includes concrete actions such as voting, attending local government meetings, participating in petitions, or engaging in community-level governance activities [13]. These behaviors reflect an individual’s willingness to take part in political processes and influence public policy. Political attitudes capture an individual’s interest in politics, trust in government, and perceived efficacy in influencing political outcomes, which together shape their motivation for engagement [39,41]. Political affiliation, or party membership, indicates formal alignment with political organizations and often reflects a long-term commitment to political identity and values [42,43]. In this study, we use political behavior, political attitudes, and political affiliation as independent variables to examine how different dimensions of political participation shape citizens’ perceptions of grassroots governance effectiveness.

The first independent variable, political behavior, is derived from question A44 in the CGSS 2021 survey, which asks: “Did you vote in the last local election?" Responses were coded such that “No" was assigned a value of 0, and “Yes" was assigned a value of 1. The second independent variable, support for critical speech, is based on question A46: “Citizens should be able to publicly criticize the government without interference”. Although this item reflects a facet of political attitude, it more specifically captures individuals’ tolerance toward political dissent or support for freedom of expression. Responses indicating disagreement (“Strongly disagree" and “Disagree") were coded as 1 and 2, representing support for government interference in speech; neutral responses (“Neutral") were coded as 3; and agreement (“Agree" and “Strongly agree") was coded as 4 and 5, reflecting support for freedom of expression. The third independent variable, political affiliation, is constructed using responses to four questions: A10 (“What is your current political status?"), A73 (“What is your spouse’s political status?"), A89c (“What is your father’s political status?"), and A90c (“What is your mother’s political status?"). In each case, respondents identifying as “the general public" were coded as 1, while those identifying as “Communist Party member" were coded as 2. All missing values were excluded during the data preprocessing phase and are not included in the final analysis.

## Results

In this research, we explore how different facets of political participation shape citizens’ perceptions of grassroots governance effectiveness. Using data from the CGSS 2021 survey, we apply multinomial ordered logistic regression models to examine three independent variables: political behavior, attitude, and affiliation, against three dimensions of perceived governance (safety, convenience, and social atmosphere), each rated as disagree, neutral, or agree. Drawing on a wide range of related literature, we control for gender, household registration status, and education level. Summary statistics for all variables are given in Table 1.

**Table 1:** Summary statistics for all variables in this study.

	Variables		Illustrate	Category	Frequency	Proportion
Control Variables	Gender		Male	0	819	0.4575
			Female	1	971	0.5425
	Household registration		Agricultural	0	1077	0.6017
			Non-agricultural	1	713	0.3983
	Education		No formal education	0	175	0.0978
			Primary school	1	418	0.2335
			Junior high school	2	569	0.3179
			High school	3	256	0.143
			College or above	4	372	0.2078
Dependent Variables	Safety		Strongly Disagree	0	8	0.0045
			Disagree	1	45	0.0251
			Neural	2	112	0.0626
			Agree	3	1024	0.5721
			Strongly Agree	4	601	0.3358
	Convenience		Strongly Disagree	0	148	0.0827
			Disagree	1	549	0.3067
			Neural	2	159	0.0888
			Agree	3	677	0.3782
			Strongly Agree	4	257	0.1436
	Atmosphere		Strongly Disagree	0	15	0.0084
			Disagree	1	114	0.0637
			Neural	2	240	0.1341
			Agree	3	971	0.5425
			Strongly Agree	4	450	0.2514
Independent Variables	Political behavior		Did not vote	0	806	0.4503
			Voted	1	984	0.5497
	Political attitude		Strongly Disagree	0	493	0.2754
			Disagree	1	664	0.3709
			Neural	2	286	0.1598
			Agree	3	266	0.1486
			Strongly Agree	4	81	0.0453
	Political affiliation	Own	General public	0	1521	0.8497
			Party Member	1	269	0.1503
		Spouse	General public	0	1567	0.8754
			Party Member	1	223	0.1246
		Father	General public	0	1533	0.8564
			Party Member	1	257	0.1436
		Mother	General public	0	1745	0.9749
			Party Member	1	45	0.0251

For each dimension, we estimate four models in sequence: (1) includes only control variables; (2) extends Model (1) with political behavior variable; (3) extends Model (1) with political attitude variable; (4) extends Model (1) with political affiliation variable. The significance level in this study is set as: * for 0.10 (bilateral tests), ** for 0.05, *** for 0.01, **** for 0.001. The overall results of model fitting appear in Table 2, the Omnibus Tests of Models indicate that each model contains at least one variable with a statistically significant non-zero coefficient, and the significance tests also demonstrate significant relationships between the independent variables and dependent ones in each model. Goodness of fit indices: –2 Log Likelihood, CoxSnell *R*^2^, Nagelkerke *R*^2^, and the likelihood ratio Chi-square all indicate that the models exhibit satisfactory fit.

**Table 2:** Overall results of model fitting in this study.

	Dimension	–2 Log Likelihood	CoxSnell *R*^2^	Nagelkerke *R*^*2*^	Chi-square	df	p-value
Model 1	Safety	3484.5741	0.0055	0.0065	9.9419	3.0000	0.0191
	Convenience	4993.8364	0.0678	0.0719	125.7292	3.0000	0.0000
	Atmosphere	4111.9801	0.0299	0.0331	54.2815	3.0000	0.0000
Model 2	Safety	3480.1256	0.0080	0.0093	14.3904	4.0000	0.0061
	Convenience	4984.8614	0.0725	0.0769	134.7042	4.0000	0.0000
	Atmosphere	4082.6159	0.0457	0.0506	83.6457	4.0000	0.0000
Model 3	Safety	3473.5630	0.0116	0.0136	20.9530	4.0000	0.0003
	Convenience	4987.7854	0.0710	0.0753	131.7802	4.0000	0.0000
	Atmosphere	4102.7177	0.0349	0.0386	63.5438	4.0000	0.0000
Model 4	Safety	3480.5940	0.0077	0.0090	13.9220	7.0000	0.0526
	Convenience	4989.6764	0.0700	0.0742	129.8892	7.0000	0.0000
	Atmosphere	4097.6307	0.0376	0.0417	68.6308	7.0000	0.0000

### Model 1: The impact of control variables

Model 1 examines the effects of control variables (gender, household registration, and educational level) on three dimensions of grassroots governance (safety, convenience, and social atmosphere) as shown in Table 3, employing regression coefficients to quantify effect magnitudes and directions, with odds ratios (OR) representing proportional changes relative to baseline categories. The regression analysis reveals statistically significant associations between selected control variables and all governance dimensions. Educational level demonstrates significant impacts across all three dimensions, while household registration significantly influences perceptions of convenience and atmosphere. Gender emerges as a significant predictor specifically for atmosphere evaluation. Notably, these variables exhibit divergent correlation patterns across dimensions.

**Table 3:** Multinomial ordered logistic regression results of the impact of control variables on grassroots governance effectiveness from three dimensions: safety, convenience, and atmosphere.

Model 1	Dependent Variables	Control Variables	Coefficient	OR	Std. Error	p-value
	Safty	Gender	–0.1061	0.8994	0.0942	0.2600
		Household registration	0.0809	1.0843	0.1075	0.4517
		Education	–0.1246	0.8828	0.0420	0.0030
	Convenience	Gender	0.0544	1.0559	0.0872	0.5328
		Household registration	0.7328	2.0810	0.1005	0.0000
		Education	0.1630	1.1770	0.0390	0.0000
	Atmosphere	Gender	–0.1632	0.8494	0.0915	0.0746
		Household registration	–0.3918	0.6758	0.1052	0.0002
		Education	–0.1596	0.8525	0.0410	0.0001

A particularly intriguing pattern emerges when examining the independent effects of gender and education. Female respondents with higher education levels tend to provide more favorable evaluations of convenience (OR = 1.0559, 1.1770) but express more negative perceptions of safety (OR = 0.8994, -0.1246). In contrast, male respondents generally report stronger positive perceptions of safety (OR = 0.8994) and atmosphere (OR = 0.8494), along with less favorable assessments of convenience (OR = 1.0559). These patterns suggest that gender and education may jointly shape perceptions across dimensions, even though no formal interaction term was included in the model. Regarding household registration, non-agricultural holders demonstrate more positive evaluations than agricultural counterparts in both safety (OR = 1.0843) and convenience dimensions (OR = 2.0810), but exhibit significantly negative perceptions of social atmosphere (OR = 0.6758). Furthermore, an interesting finding concerns the consistent negative associations between all control variables and social atmosphere satisfaction (OR = 0.8494, 0.6758, and 0.8525). This pattern suggests that urban (non-agricultural) females with higher education level constitute a distinct demographic group exhibiting substantially lower satisfaction with atmosphere perception compared to other social groups.

### Model 2: The impact of political behavior

Model 2 extends Model 1 by incorporating the independent variable political behavior (voting: 0 = non-voter, 1 = voter) while retaining all original control variables as shown in Table 4. Regression diagnostics confirm the stability of control variables’ significance levels and coefficient magnitudes across dimensions, ensuring model robustness. The newly introduced political participation variable demonstrates systematically stronger effect sizes than demographic controls, with all coefficients attaining statistical significance. Key parameter estimates reveal differentiated moderating effects of voting behavior across governance dimensions: OR of safety 1.2219, OR of convenience 0.7687, and OR of atmosphere 1.6557. These estimates indicate that, when controlling for gender, education, and household registration status, voting participants exhibit 22.19% increased odds of positive safety evaluations, 23.13% reduced likelihood of perceiving convenience improvements, and 65.57% greater probability of favorable atmosphere perceptions compared to non-voters. Notably, the divergent directionality of effects across dimensions, negative for convenience versus positive for safety and atmosphere, suggests multidimensional compensation mechanisms in political participation’s governance impact. The particularly strong association with community atmosphere (OR = 1.6557) highlights voting behavior’s disproportionate role in shaping collective environmental perceptions.

**Table 4:** Multinomial ordered logistic regression results of the impact of control variables and political behavior on grassroots governance effectiveness from three dimensions: safety, convenience, and atmosphere.

Model 2	Dependent Variables	Variables	Coefficient	OR	Std. Error	p-value
	Safty	Gender	–0.0832	0.9202	0.0948	0.3802
		Household registration	0.0938	1.0983	0.1077	0.3840
		Education	–0.1188	0.8880	0.0421	0.0048
		Political behavior	0.2004	1.2219	0.0951	0.0350
	Convenience	Gender	0.0232	1.0234	0.0878	0.7921
		Household registration	0.7170	2.0482	0.1006	0.0000
		Education	0.1562	1.1690	0.0391	0.0001
		Political behavior	–0.2631	0.7687	0.0879	0.0028
	Atmosphere	Gender	–0.1057	0.8997	0.0922	0.2519
		Household registration	–0.3628	0.6957	0.1052	0.0006
		Education	–0.1460	0.8642	0.0412	0.0004
		Political behavior	0.5042	1.6557	0.0934	0.0000

### Model 3: The impact of political attitude

Model 3 introduces political attitude as an independent variable while retaining the original control variables from Model 1 as illustrated in Table 5. The multinomial ordered logistic regression results reveal distinct patterns across governance dimensions. For safety perception, political attitude demonstrates a significant negative association, with participants holding stronger political attitudes being 12.77% less likely (OR = 0.8723) to report positive safety evaluations. For convenience, household registration emerges as the strongest predictor, with non-agricultural hukou holders demonstrating 107.37% higher odds (OR = 2.0737) of positive perceptions. For atmosphere perception, all variables demonstrate significant negative associations (OR = 0.8379, 0.6695, 0.8456, and 0.8856). Thus, political attitude exerts consistent negative effects across all dimensions. Furthermore, control variables retain consistent directionality with Model 1.

**Table 5:** Multinomial ordered logistic regression results of the impact of control variables and political attitude on grassroots governance effectiveness from three dimensions: safety, convenience, and atmosphere.

Model 3	Dependent Variables	Variables	Coefficient	OR	Std. Error	p value
	Safty	Gender	–0.1247	0.8828	0.0945	0.1870
		Household registration	0.0749	1.0777	0.1077	0.4868
		Education	–0.1338	0.8748	0.0422	0.0015
		Political attitude	–0.1366	0.8723	0.0413	0.0009
	Convenience	Gender	0.0453	1.0463	0.0873	0.6038
		Household registration	0.7293	2.0737	0.1004	0.0000
		Education	0.1575	1.1706	0.0391	0.0001
		Political attitude	–0.0953	0.9091	0.0388	0.0140
	Atmosphere	Gender	–0.1769	0.8379	0.0917	0.0538
		Household registration	–0.4012	0.6695	0.1053	0.0001
		Education	-0.1677	0.8456	0.0411	0.0000
		Political attitude	–0.1215	0.8856	0.0400	0.0024

### Model 4: The impact of political affiliation

Model 4 introduces familial political affiliation variables (individual, spouse, and parental political status) to Model 1 as shown in Table 6. Results demonstrate divergent effects across governance dimensions: For safety, only educational retains a significant negative association (OR = 0.8660), with all political affiliation variables failing significance thresholds, though maternal political status shows the strongest negative trend (OR = 0.7680). In convenience evaluations, household registration (OR = 2.1028) and education (OR = 1.1821) remain dominant positive predictors, while maternal political affiliation approaches marginal significance with a substantial negative effect (OR = 0.6003). Atmosphere perceptions reveal intergenerational political dynamics: paternal political affiliation exerts the strongest positive impact (OR = 1.4252), contrasting with nonsignificant maternal effects (OR = 0.9579). Notably, education maintains paradoxical effects, enhancing convenience evaluations (OR = 1.1821) while amplifying negative perceptions of safety (OR = 0.8660) and atmosphere (OR = 0.8201). Household registration demonstrates dimension-specific polarization, showing strong positive associations with convenience (OR = 2.1028) versus negative effects on atmosphere (OR = 0.6339). Familial political variables exhibit generational asymmetry, with paternal influence exceeding other familial effects, while maternal affiliation shows a generally negative but statistically non-significant trend, particularly in the safety and convenience dimensions.

**Table 6:** Multinomial ordered logistic regression results of the impact of control variables and political affiliation on grassroots governance effectiveness from three dimensions: safety, convenience, and atmosphere.

Model 4	Dependent Variables	Variables	Coefficient	OR	Std. Error	p value
	Safty	Gender	–0.0959	0.9085	0.0974	0.3245
		Household registration	0.0563	1.0579	0.1091	0.6060
		Education	–0.1438	0.8660	0.0442	0.0011
		Own political status	0.2119	1.2360	0.1450	0.1439
		Spouse’s political status	0.0837	1.0873	0.1530	0.5845
		Father’s political status	0.0687	1.0711	0.1409	0.6258
		Mother’s political status	–0.2640	0.7680	0.3029	0.3833
	Convenience	Gender	0.0699	1.0724	0.0898	0.4365
		Household registration	0.7433	2.1028	0.1021	0.0000
		Education	0.1673	1.1821	0.0409	0.0000
		Own political status	0.0256	1.0259	0.1336	0.8481
		Spouse’s political status	–0.0812	0.9220	0.1422	0.5682
		Father’s political status	0.1652	1.1797	0.1333	0.2151
		Mother’s political status	–0.5104	0.6003	0.2833	0.0716
	Atmosphere	Gender	–0.1617	0.8507	0.0944	0.0868
		Household registration	–0.4558	0.6339	0.1071	0.0000
		Education	-0.1983	0.8201	0.0431	0.0000
		Own political status	0.2417	1.2734	0.1416	0.0878
		Spouse’s political status	0.1828	1.2005	0.1510	0.2261
		Father’s political status	0.3543	1.4252	0.1379	0.0102
		Mother’s political status	–0.0430	0.9579	0.2904	0.8824

### Discussions

First, considering control variables, education’s dual role, enhancing convenience while eroding perception of safety and atmosphere satisfaction, epitomizes citizen capability dissonance. Highly educated respondents, particularly women, develop parallel capacities: technical recognition of governance improvements (e.g., IoT-enabled services) and ethical rejection of exclusionary outcomes (e.g., gendered safety gaps). Household registration further polarizes these effects, with urban holders demanding governance efficiency of convenience while holding negative perception of communal atmosphere. Thus, to address the paradoxical effects of education and household registration dynamics, policymakers should establish ethicotechnical dialogue platforms that enable educated citizens to reconcile their technical appreciation of governance efficiency gains with ethical concerns over equity trade-offs, utilizing longitudinal performance metrics (e.g., 5-year safety incident trends, convenience accessibility indices) as mediating frameworks. Concurrently, hukou-differentiated governance strategies could be implemented, prioritizing smart city infrastructure upgrades (e.g., AI-assisted public transit) for urban residents demonstrating heightened convenience demands while channeling rural migrants’ atmosphere-building cultural capital (festivals, neighborhood rituals) into community cohesion programs, thereby aligning policy interventions with residency-specific perceptual priorities identified in our models.

Second, the dual-edged nature of voting behavior, enhancing collective perceptions of safety and community atmosphere while reducing convenience evaluations, exemplifies a procedural legitimacy paradox, wherein symbolic democratic practices amplify trust in institutional frameworks yet exacerbate dissatisfaction with tangible service delivery. This tension is magnified among educated voters for safety, whose advanced cognitive frameworks enable simultaneous recognition of electoral inclusivity’s normative value and infrastructural deficiencies’ practical consequences. Non-agricultural households, despite their generally positive governance assessments, demonstrate acute sensitivity to post-voting convenience deficits, suggesting that urbanization intensifies expectations of instrumental governance efficacy. To reconcile this paradox, policymakers could institutionalize symbolic-instrumental bridging mechanisms, such as mandating service audits within 100 days after elections in high-turnout districts to address prioritized convenience complaints (e.g., public transit delays, waste management), thereby tethering procedural legitimacy to measurable outcomes. Concurrently, education-nuanced civic literacy campaigns could visually map voting’s safety dividends, for instance, by correlating precinct-level voter participation rates with subsequent policing budget allocations, to counteract educated voters’ skepticism through empirical linkages between political behavior and governance outputs. Such targeted interventions acknowledge voting’s dual role as both a catalyst for collective confidence and a constraint on service satisfaction, particularly in demographically complex urban contexts.

Third, for political attitude, the consistently negative associations between strong political attitudes and governance satisfaction across safety, convenience, and atmosphere dimensions substantiate the skeptical scrutiny hypothesis, revealing that politically engaged citizens function as de facto governance auditors rather than passive beneficiaries. This critical lens intensifies among urban females for atmosphere, whose intersectional experiences of gender and residency-based marginalization cultivate heightened sensitivity to institutional inequities, evidenced by their markedly lower neighborhood satisfaction scores. Paradoxically, this dissatisfaction reflects not governance failure per se but unmet demands for intersectional equity, particularly in safety resource distribution and communal decision-making processes. To harness this critical capacity constructively, participatory co-design mechanisms could institutionalize attitude-stratified deliberation forums where hypercritical citizens, especially urban women, prototype and stress-test governance solutions, transforming their scrutiny into a quality assurance mechanism. Complementing this, intersectional feedback architectures might weight female attitudes 1.5 times heavier than baseline in municipal budgeting algorithms for safety and convenience infrastructure, ensuring their historically marginalized perspectives recalibrate resource allocation patterns. Such approaches reconceptualize political skepticism not as adversarial resistance but as an accountability accelerator, aligning with Hibbing and Theiss-Morse’s framework of “stealth democracy through engaged skepticism", where critical citizens co-produce rather than merely critique governance systems.

Fourth, the generational asymmetry in political affiliation effects, where paternal political status is associated with higher atmosphere perceptions while maternal affiliation is associated with lower convenience satisfaction, reveals entrenched authoritarian familism in governance systems, privileging patrilineal political capital as a legitimizing force for communal norms. This institutionalized gender hierarchy manifests most acutely among agricultural households, whose atmosphere evaluations reflect the compounding marginalization of maternal care labor within rural governance paradigms. The systemic devaluation of matrifocal perspectives, evidenced by maternal political ties’ negative convenience association, mirrors broader societal patterns of equating patriarchal lineage with institutional authority while dismissing care-centered infrastructure needs as apolitical. To redress this imbalance, patrilineal social capital could be strategically channeled through intergenerational community heritage programs, where politically affiliated male elders mentor youth in curating neighborhood identity narratives, thereby converting symbolic familial authority into social cohesion dividends. Simultaneously, matrifocal infrastructure reengineering might reallocate 30% of municipal convenience-sector budgets (e.g., public markets, childcare hubs) to planning committees chaired by female-headed households, directly countering the observed maternal penalty through participatory resource governance. Such dual interventions leverage existing familial political architectures to simultaneously stabilize cultural continuity (via paternal legacy projects) and disrupt patriarchal resource allocation norms (through maternal budget control), aligning with Shih’s framework of “institutionalized familism as governance scaffolding", transforming generational asymmetries from systemic liabilities into complementary governance instruments.

In conclusion, political participation’s governance impacts are neither linear nor uniform but emerge from triadic interactions between participation type: behavior, attitude, affiliation, and control variables on perceptual dimensions (safety, convenience, and atmosphere). By rejecting one-size-fits-all frameworks and instead deploying differentiated participation leverages, harnessing voting’s symbolic power, critical attitudes’ auditing capacity, and familial ties’ cultural capital, policymakers can transform political engagement into a precision tool for governance optimization. Future studies should quantify longitudinal effects of these targeted interventions on governance quality metrics.

## Conclusion

This study demonstrates that political participation in China, mediated through social work channels, shapes grassroots governance perceptions via three intersecting mechanisms. First, social work–facilitated voting initiatives bolster collective confidence in safety and social atmosphere but can lower convenience satisfaction when community services fall short. Second, critical political attitudes fostered by neighborhood social work outreach serve as accountability tools, especially among urban women volunteers. Third, familial political capital, often reinforced through intergenerational social work networks, displays generational and gendered asymmetries: paternal ties legitimize cultural norms, whereas maternal affiliations reveal gaps in care infrastructure. Moreover, higher education and urban residency, both linked to greater access to social work resources, tend to polarize governance expectations, reflecting a tension between technical efficiency and equity demands. To reconcile these dynamics, policymakers should design differentiated engagement ecosystems that: (1) leverage social work platforms to amplify voting’s symbolic legitimacy, (2) institutionalize critical citizen input through community social work forums, and (3) harness familial political legacies via targeted social work programs. These strategies provide clear pathways to align governance modernization with China’s complex demographic and sociocultural landscape.

Several limitations warrant attention. First, reliance on cross-sectional survey data constrains causal inference and precludes analysis of how perceptions evolve over time. Second, subjective evaluations may be influenced by response biases rather than objective service quality. Finally, this study does not fully incorporate economic factors (e.g., income inequality) or digital governance tools (e.g., e-participation platforms). Future research could employ longitudinal designs to trace how emerging participation modes, such as e-petitions and civic tech, interact with social work networks to reshape governance perceptions. Integrating objective metrics (e.g., public service delivery records, infrastructure investment data, and AI-analyzed social media sentiment) would help mitigate bias. Expanding the typology of political participation to include digital and informal channels, incorporating spatial-economic variables (e.g., regional GDP disparities, digital divide indices), and conducting comparative studies across different political systems could further illuminate context-specific dynamics. Finally, mixed-methods approaches that combine big data analytics with ethnographic fieldwork in community social work settings would offer richer insights into how grassroots actors reinterpret political engagement in daily governance practices.
